# Developmental dynamics of the neural crest–mesenchymal axis in creating the thymic microenvironment

**DOI:** 10.1126/sciadv.abm9844

**Published:** 2022-05-13

**Authors:** Adam E. Handel, Stanley Cheuk, Fatima Dhalla, Stefano Maio, Tania Hübscher, Ioanna Rota, Mary E. Deadman, Olov Ekwall, Matthias Lütolf, Kenneth Weinberg, Georg Holländer

**Affiliations:** 1 Department of Paediatrics and the Weatherall Institute of Molecular Medicine, University of Oxford, Oxford, UK.; 2 Nuffield Department of Clinical Neurosciences, University of Oxford, Oxford, UK.; 3 Department of Rheumatology and Inflammation Research, University of Gothenburg, Gothenburg, Sweden.; 4 Laboratory of Stem Cell Bioengineering, Swiss Federal Institute of Technology in Lausanne, Lausanne, Switzerland.; 5 Department of Pediatrics, University of Gothenburg, Gothenburg, Sweden.; 6 Division of Stem Cell Transplantation and Regenerative Medicine Department of Pediatrics, Stanford University, Stanford, CA, USA.; 7 Paediatric Immunology, Department of Biomedicine, University of Basel and University Children’s Hospital Basel, Basel, Switzerland.; 8 Department of Biosystems Science and Engineering, ETH Zurich, Basel, Switzerland.

## Abstract

The thymic stroma is composed of epithelial and nonepithelial cells providing separate microenvironments controlling homing, differentiation, and selection of hematopoietic precursor cells to functional T cells. Here, we explore at single-cell resolution the complex composition and dynamic changes of the nonepithelial stromal compartment across different developmental stages in the human and mouse thymus, and in an experimental model of the DiGeorge syndrome, the most common form of human thymic hypoplasia. The detected gene expression signatures identify previously unknown stromal subtypes and relate their individual molecular profiles to separate differentiation trajectories and functions, revealing an unprecedented heterogeneity of different cell types that emerge at discrete developmental stages and vary in their expression of key regulatory signaling circuits and extracellular matrix components. Together, these findings highlight the dynamic complexity of the nonepithelial thymus stroma and link this to separate instructive roles essential for normal thymus organogenesis and tissue maintenance.

## Teaser

Single-cell profiling of thymic stroma identifies a dynamic contribution from neural crest cells to the thymic mesenchyme.

## INTRODUCTION

Thymic T cell lineage commitment, development, maturation, and repertoire selection are instructed by a stromal scaffold that includes endothelial cells and mesenchymal cells. The thymic epithelial cell (TEC) (*1*) compartment is both phenotypically and transcriptionally well characterized, providing at single-cell resolution a detailed account of the cells’ developmental dynamics and functions (*2*, *3*). In addition, the thymus microenvironment is also composed of stromal cells of mesenchymal origin, including fibroblasts (*4*, *5*), endothelial cells (*6*), and vascular mural cells (*7*). Derived primarily from either mesoderm or ectodermal neural crest cells, these thymic mesenchymal cells interact with TEC and thus create unique cellular niches that control thymopoiesis. This critical function of mesenchymal cells is accomplished via the production of extracellular matrix components, morphogens, and key growth factors (*4*, *5*, *8*). Hence, the thymic mesenchyme is indispensable for the organ’s correct formation and function (*1*, *8*, *9*).

Fibroblasts constitute the largest component of the nonepithelial thymic stroma (NETS). Although first described as a distinct cell type over 150 years ago, the specific contributions of fibroblasts to organ formation, maintenance, and function have only recently begun to be unraveled (*9*). Using single-cell genomic technologies for the comparison of diverse tissues, fibroblasts were noted to display substantial heterogeneity with both cross-organ communalities and tissue-specific differences (*10*). Likewise, endothelial cells and vascular mural cells display organotypic features that have only recently been appreciated when resolving the cells’ distinct transcriptomes at single-cell resolution (*11*). In addition to their essential role in providing oxygen, nutrients, cells, and other cargo to tissues, blood vessels also express in a context-specific fashion diverse transcriptomic profiles that include sets of growth factors inducing, specifying, patterning, and guiding organ formation and homeostasis (*12*). A third stromal component of nonepithelial origin is neural crest cells, which enter the anlage as a migratory population as early as embryonic day (E) 12 where they differentiate into distinct cell types, including vasculature-associated pericytes juxtaposed between endothelia and the other components of the stromal scaffold (*7*).

A detailed phenotypic, transcriptomic, and functional genomic description of the diverse population of NETS cells is to date still wanting. We have therefore used flow cytometry and single-cell multiomics technologies to detail the complexity and developmental dynamics of thymic mesenchymal cells in both mouse and human tissue. Our results highlight a previously unappreciated heterogeneity among cells belonging to the NETS under physiological conditions and identify distinct yet selective defects of these cells in a genetic mouse model of the 22q11 deletion syndrome, the most common human condition associated with congenital thymus hypoplasia.

## RESULTS

### Single-cell sequencing reveals high levels of complexity within the thymic mesenchyme

**Fig. 1.**
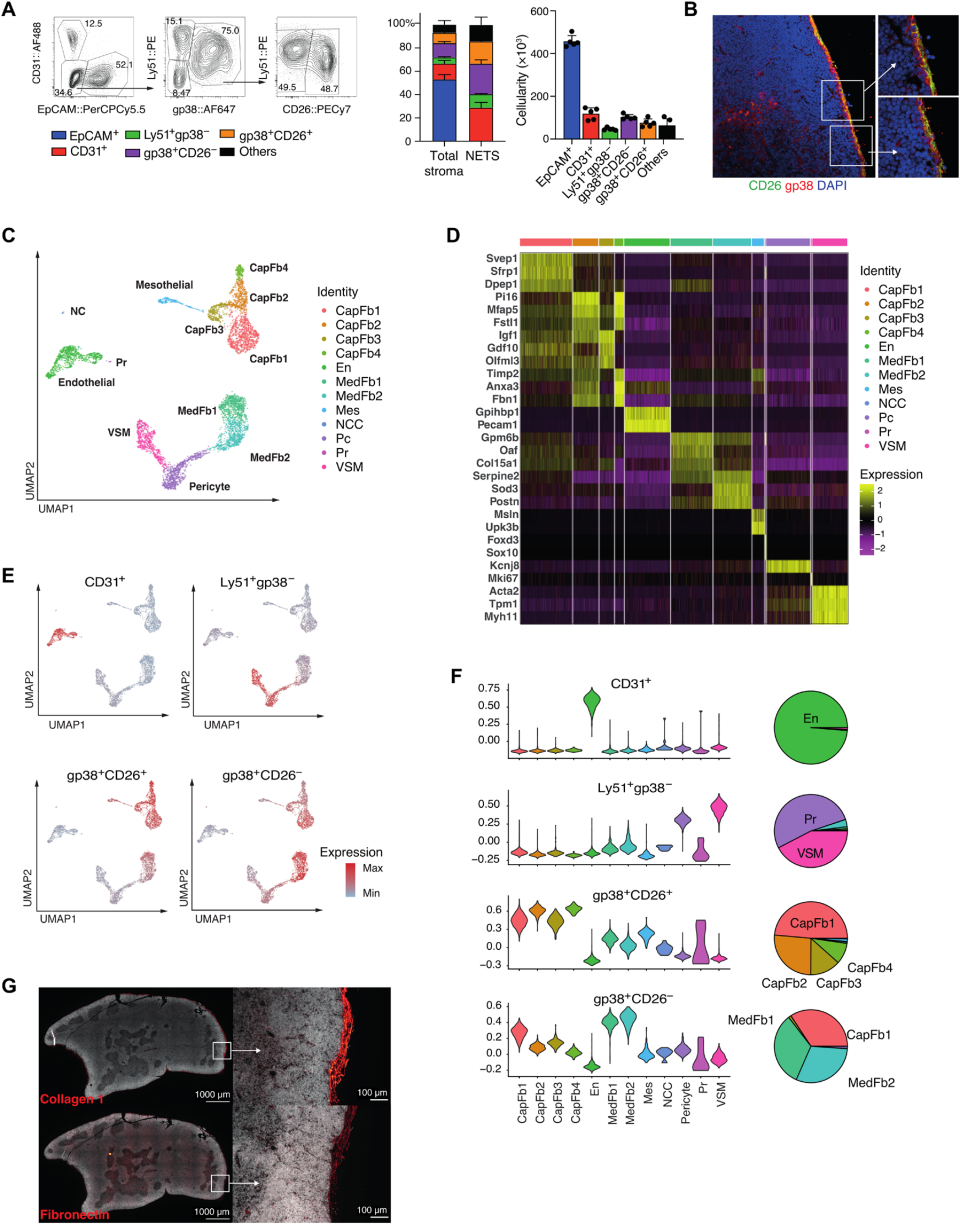
Heterogeneity of the thymic stroma at 4 weeks of age. (**A**) Representative FACS plot of live Ter119^−^CD45^−^EpCAM^−^ thymic stromal cells at 4 weeks old (left), the relative frequency (middle), and cellularity (right) of CD31^+^, Ly51^+^gp38^−^, gp38^+^CD26^−^, and gp38^+^CD26^+^ cells among total stroma or nonepithelial stroma. (**B**) Immunofluorescence staining of thymic mesenchymal cells [red, gp38; green, CD26; blue, 4′,6-diamidino-2-phenylindole (DAPI)]. (**C**) UMAP plot of Ter119^−^CD45^−^EpCAM^−^ cells from 4-week-old mice. (**D**) Heatmap of top five differentially expressed genes between each cluster. (**E**) Overlay on UMAP plot and (**F**) violin plots of the expression of genes specific to each FACS-isolated subpopulation from bulk RNA sequencing (RNA-seq) and pie charts showing the proportion of cell types that express gene signatures characteristic of specific FACS-isolated populations as inferred from bulk RNA-seq. CapFb, capsular fibroblast; En, endothelium; MedFb, medullary fibroblast; Mes, mesothelium; NCC, neural crest; Pc, pericyte; Pr, proliferating cell; VSM, vascular smooth muscle. (**G**) Immunofluorescence microscopy showing the distribution of type I collagen and fibronectin in a 5-week-old mouse thymus. FACS data shown in (A) were representative of one experiment (*n* = 5) out of two independent experiments (total *n* = 7), and mean value and SD are shown in the corresponding bar graphs (*n* = 5).

We first sought to delineate both the frequency and diversity of NETS cells (phenotypically defined as Ter119^−^CD45^−^EpCAM^−^) in the thymus of 4-week-old mice. These cells accounted for approximately half of the total thymic stroma cellularity, and distinct subpopulations were identified using the differential expression of glutamyl aminopeptidase Ly51, glycoprotein podoplanin (gp38), dipeptidyl peptidase-4 (DPP4; CD26), and platelet endothelial cell adhesion molecule (PECAM1; CD31) (Fig. 1A and fig. S1A) (*4*, *13*). The Ly51^hi^gp38^−^ phenotype identified neural crest–derived pericytes that surround blood vessels adjacent to CD31^+^ endothelial cells (*5*, *7*). The gp38-positive stromal cells expressed a reduced level of Ly51 and could be further differentiated into separate subpopulations based on their CD26 expression: gp38^+^CD26^+^ cells were localized to the thymus capsule, whereas gp38^+^CD26^−^ cells were enriched in the medulla (Fig. 1B) (*13*).

To delineate the heterogeneity of the thymic mesenchyme in an unbiased fashion and independent of a limited number of phenotypic markers, we next generated transcriptomic libraries from 5878 single Ter119^−^CD45^−^EpCAM^−^ cells isolated from 4-week-old thymi (fig. S1A). We identified 12 distinct cell subtypes based on their separate gene expression profiles (Fig. 1, C and D, and fig. S1B). (For clarity, we refer to transcriptionally defined stromal cell clusters as subtypes, whereas the terms populations and subpopulations specify cells that have been defined by cytometry.) A Uniform Manifold Approximation and Projection (UMAP) analysis of non-TEC stroma identified four separate cell clusters of different sizes. The two largest comprised several individual cell subtypes that were transcriptomically defined as either capsular or medullary fibroblasts (Fig. 1C and fig. S1C) (*13*). The capsular cluster closely resembling the gene expression profile of capsular fibroblasts consisted of four subsets that separated from mesothelial cells defined—inter alia—by their expression of *Msln* and *Upk3b*, encoding the glycosylphosphatidylinositol-anchored cell surface adhesion protein mesothelin and the membrane integral protein uroplakin, respectively (*14*). Within the capsular fibroblast clusters, subtype 1 (designated CapFb1) was characterized by the high expression of *Svep1*, *Sfrp1*, and *Dpep1*, which encode a multidomain cellular adhesion molecule (*15*), the secreted frizzled-related protein 1 modulating stromal to epithelial signaling via Wnt inhibition (*16*), and a membrane-bound dipeptidase involved in the metabolism of glutathione and other similar proteins (*17*). The capsular subtypes 2 (CapFb2) and 4 (CapFb4) were characterized by the expression of *Pi16*, *Mfap5*, and *Fstl1*, which encode a peptidase inhibitor of largely unknown function, the microfibrillar-associated protein 5 related to extracellular matrix remodeling and inflammation (*18*), and the secreted extracellular glycoprotein follistatin-like 1. CapFb4 also highly expressed *Timp2* encoding the tissue inhibitor of metalloproteinase 2 relevant for tissue remodeling (*19*), *Anxa3* translating into the membrane-associated annexin 3 protein activating the epithelial-to-mesenchymal transition (EMT) program and Wnt signaling pathway (*20*), and *Fbn1* encoding fibrillin 1, a major component of extracellular microfibrils. The CapFb3 subtype typically expressed *Igf1* encoding insulin growth factor 1 regulating tissue homeostasis via cell proliferation, differentiation, maturation, and survival (*21*); *Gdf10* translating into the transforming growth factor–β (TGF-β) superfamily member growth differentiation factor 10 (GDF10) (*22*); and *Olfml3* encoding the secreted glycoprotein olfactomedin-like 3 that has matrix-related functions central to embryonic development (*23*).

The second UMAP cluster incorporated two distinct medullary fibroblast subtypes, pericytes and vascular smooth muscle cells. The medullary fibroblast subtypes 1 (MedFb1) and 2 (MedFb2) displayed similar gene expression profiles, although transcripts for the out-at-first protein (encoded by *Oaf*) and the α1 chain of collagen XV (*Col15a1*) were detected at higher levels in MedFb1, while transcripts for extracellular superoxide dismutase 3 (*Sod3*) were particularly evident in MedFb2. Both subtypes also comprised transcripts for interleukin-33 (IL-33) and *Cxcl16*, which are important for dendritic cell activation and natural killer T (NKT) cell migration, respectively (*24*, *25*). Transcripts related to antigen processing and presentation were enriched in MedFb2 (fig. S1B). However, contrary to a recent observation (*13*), tissue-restricted antigens were not generally more frequent in medullary fibroblasts when compared to other NETS (fig. S1E).

Pericytes (Pc) were identified by their characteristic expression of *Kcnj8* encoding member 8 of the J subfamily of the potassium inwardly rectifying channels, which form part of the adenosine triphosphate (ATP)/adenosine diphosphate (ADP)–binding potassium channel of these cells (*26*). Vascular smooth muscle cells (VSM) were characterized by the expression of contractile elements, including *Acta2*, *Tpm1*, and *Myh11*, whereas endothelial cells displayed a high number of transcripts for *Gpihbp1* and *Pecam1*, which encode the glycosylphosphatidylinositol-anchored high-density lipoprotein binding protein 1 and the intercellular junction protein platelet and endothelial cell adhesion molecule, respectively.

Neural crest–derived cells (NCCs) were characterized by their expression of *Foxd3* and *Sox10*, which are critical for the cells’ specification and development (*27*, *28*). *Sox10* expression was highly NCC specific (fig. S2). Other members of the SOX transcription factor family showed particular expression patterns in certain cell types, e.g., *Sox6* was detected in NCC, VSM, and mesothelium, whereas *Sox9* was found in CapFb3, and *Sox17/Sox18* in endothelia. Last, actively proliferating cells (Pr) were identified by the expression of different cell cycle–related genes including *Mki67* encoding the nuclear protein Ki67.

We determined the gene expression profiles of the four fluorescence-activated cell sorting (FACS)–defined non-TEC thymic stromal subpopulations (fig. S1A) and deconvoluted the individual transcriptomes by projection onto the single-cell UMAP data (Fig. 1E and Table 1). Stroma cells expressing CD31 identified the cluster defined as endothelial cells, and the Ly51^+^gp38^−^ subpopulation represented the Pc and VSM clusters (Fig. 1, E and F). The gp38^+^CD26^+^ subpopulation included all of the four capsular fibroblast subtypes, whereas the gp38^+^CD26^−^ subpopulation was mainly enriched for the medullary fibroblast subtypes but also included CapFb1 cells (Fig. 1, E and F). We showed that this fibroblast heterogeneity patterned the thymic extracellular matrix by staining for two key extracellular matrix molecules (type I collagens and fibronectin), which were most highly expressed in capsular fibroblasts (Fig. 1G). We used RNA in situ hybridization to visualize one key marker each for capsular (*Pi16*) and medullary (*Csmd1*) fibroblasts, which confirmed the intrathymic localization of these subtypes (fig. S3).

**Table 1.** Thymic stromal cell types identified by single-cell RNA-seq. The three top genes (by area under the curve) and peak age are shown for each cluster. All clusters show significant differences in proportional makeup of the non-TEC thymic stromal cells across different ages (Fisher’s test with 10,000 permutations: all *P* < 0.0001).

**Cell type**	**4-week cluster**	**All ages cluster**	**Top genes**	**Peak age**
Capsular fibroblasts	CapFb1	CapFb1a	*Itm2a*, *Clec3b*, *Capn6*	E12.5
		CapFb1b	*Cdo1*, *Ptn*, *Itm2a*	P0
		CapFb1c	*Lpl*, *Thbs1*, *Mt2*	P0
	CapFb2	CapFb2a	*Adamts2*, *Mfap4*, *Bgn*	E12.5
		CapFb2b	*Col1a1*, *Col14a1*, *Mfap5*	E12.5
	CapFb3	CapFb3	*Igf1*, *Dcn*, *Cpxm1*	W4
	CapFb4	CapFb4	*Fbn1*, *Mfap5*, *Dpt*	W4
Medullary fibroblasts	MedFb1	MedFb1a	*Sele*, *Col15a1*, *Tenm4*	E13.5
		MedFb1b	*Col15a1*, *Col26a1*, *Serpine2*	W4
	MedFb2	MedFb2a	*Tmem176a*, *Des*, *Tmem176b*	W4
		MedFb2b	*Oasl2*, *Isg15*, *Iigp1*	W4
Proliferating fibroblasts	Pr	Pr	*Stmn1*, *H2afz*, *Hmgb2*	E13.5
Pericytes	Pc	Pc	*Colec11*, *Gucy1a1*, *Ebf1*	E16.5
Vascular smooth muscle	VSM	VSM	*Myh11*, *Tpm1*, *Nrip2*	W4
Neural crest cells	NCC	NCC	*Mal*, *Plp1*, *Dbi*	E12.5
Endothelium	En	EnA	*Fbln5*, *Icam2*, *Egfl8*	W4
		EnC	*Gpihbp1*, *Rgcc*, *Fabp4*	W4
		EnVL	*Selp*, *Pecam1*, *Aqp1*	W4
Mesothelium	Mes	Mes	*Upk3b*, *2010300C02Rik*, *Krt19*	W4

Hence, the single-cell RNA sequencing (RNA-seq)–based identification of thymic stromal cells unmasked a previously unrecognized heterogeneity of individual subsets among gp38^+^ NETS, which could not be identified by conventional flow cytometry–based phenotyping.

### Thymic organogenesis is characterized by dynamic mesenchymal changes

**Fig. 2.**
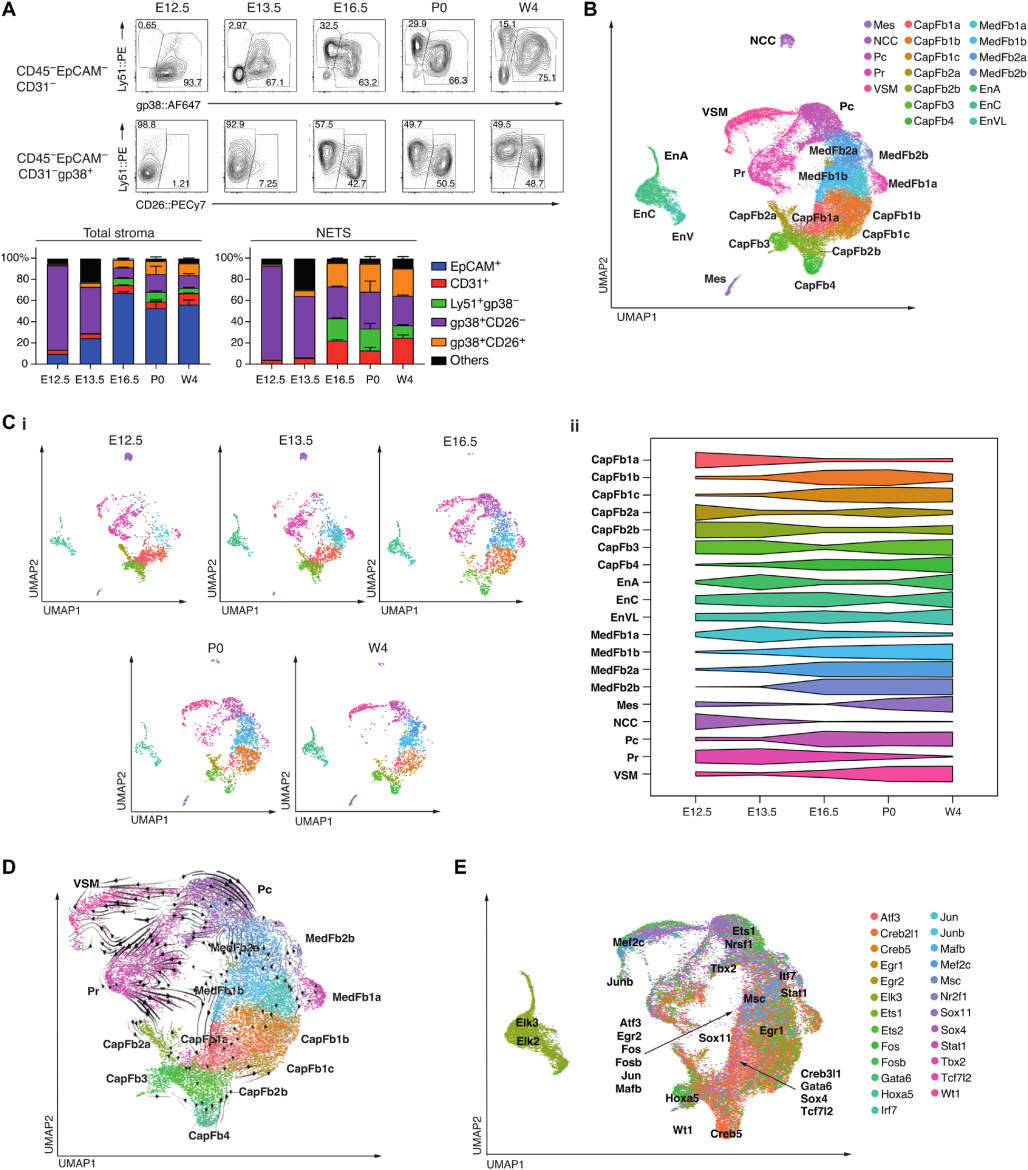
Characteristics of the thymic stroma over developmental time. (**A**) Representative FACS plots (top) of the nonepithelial thymic stroma from E12.5 to newborn (P0) and postnatal week 4 (W4), and the relative frequency (bottom) of stromal subpopulations among the total stroma (left) or the non-TEC stroma (right). (**B**) Combined UMAP plot of Ter119^−^CD45^−^EpCAM^−^ cells from mice at E12.5, E13.5, E16.5, P0, and 4 weeks of age. (**C**) (i) Individual UMAP plots for each developmental time point, with cell number down-sampled to the smallest sample size (*n* = 1997). (ii) Scaled proportional representation of each age in each cluster. (**D**) RNA velocity plot, with velocity streamlines projected onto UMAP plot. (**E**) UMAP plot, with each cell colored by the transcription factor gene regulatory network expression with the highest scaled expression. Gene regulatory network centroids are labeled. EnA, arterial endothelium; ENCC, capillary endothelium; EnVL, venous/lymphatic endothelium. E12.5 and E13.5 were sorted and analyzed from thymi pooled from two litters consisting of at least three embryos per litter. For E16.5, P0, and W4, data shown consist of cells sorted from two thymi per time point. Mean value and SD were shown in the bar charts (A).

The thymus undergoes notable microarchitectural changes during organogenesis, including the compartmentalization into distinct cortical and medullary domains and the formation of a complex vascular network (*29*). We therefore investigated how these morphological changes paralleled compositional alterations of the mesenchymal stroma (Fig. 2, A and B). At E12.5, NETS accounted for more than 90% of all CD45^−^ thymic cells, with gp38^+^CD26^−^ cells being by far the most dominant subpopulation. The frequency of TEC gradually increased parallel to thymus growth and reached a relative maximum at E16.5 when epithelia represented 60% of the thymic stroma. Earlier during thymus organogenesis, the non-TEC stroma lacked the heterogeneity observed at E16.5 and thereafter. For example, gp38^+^CD26^−^ fibroblasts dominated the stromal compartment at both E12.5 and E13.5, endothelial cells were only recognized at E13.5, and Ly51^+^gp38^−^ Pc were not detected before E16.5 (Fig. 2A and fig. S4A). The subpopulation of gp38^+^CD26^+^ capsular fibroblasts was identified as early as E13.5 and increased in frequency thereafter (Fig. 2A).

We next used single-cell RNA-seq to detail changes in the heterogeneity of individual NETS subtypes and to determine the cells’ developmental trajectories. We generated libraries on a total of 36,208 single stromal cells isolated from embryonic (E12.5, E13.5, and E16.5), newborn, and young adult thymus tissue, which collectively reiterated the clusters observed in the thymus of 4-week-old mice and provided sufficient resolution to identify additional heterogeneity (Fig. 2, B to D, and Table 1). Complex dynamic changes in the frequency of individual subtypes occurred over time between the early developmental stages and the completion of a mature thymus microenvironment. For example, CapFb1a and CapFb2b appeared early but their frequencies gradually decreased during organogenesis. Medullary fibroblasts (with the notable exception of MedFb1a) increased parallel to the emergence of medullary TEC (mTEC), suggesting that medullary fibroblasts predominantly populated the developing thymic medulla (*30*). NCCs were largely absent after E16.5, but other NETS subtypes either remained mainly unchanged or displayed a bimodal variation in frequency between E12.5 and 4 weeks of age (Fig. 2C, i and ii). This finding is in agreement with lineage tracing studies demonstrating the cells’ developmental potential to differentiate into VSM and Pc (*7*, *31*). Thus, single-cell RNA-seq revealed complex and dynamic changes in the relative number of individual NETS subtypes that would be captured incompletely by classical cell surface phenotyping, using markers such as CD26 (fig. S4B).

We leveraged the splicing information obtained from single-cell transcriptomes to determine the developmental trajectories of individual NETS subtypes. This analysis identified the CapFb1a and CapFb2b subtypes as the principal precursors for other capsular fibroblasts (Fig. 2D) and suggested MedFb1a to serve as a precursor for other fibroblast subtypes in the emerging medulla (*30*). These distinct differentiation pathways were highlighted by heterogeneity within proliferating fibroblasts, with subclusters of proliferating cells located within developmental trajectories flowing into CapFb1a, CapFb2b, MedFb1a, Pc, and VSM. This analysis also recognized CapFb3 fibroblasts as intermediates between mesothelial cells and other fibroblast subtypes, a finding consistent with the concept that fibroblasts can be derived from mesothelial cells (*32*). However, CapFb3 fibroblasts were distinct from mesothelia as they lacked the expression of *Msln* and *Upk3b* (Fig. 1D) (*14*).

We also identified age-related heterogeneity within NCCs (fig. S5). During early embryogenesis (E12.5 and E13.5), NCCs expressed genes characteristic of neuroglial identity (e.g., *Nkain4* and *Elavl3*), whereas postnatal NCCs were more likely to adopt a migratory expression profile (e.g., *Ngfr* and *Mcam*) (*33*).

The single-cell transcriptome data were also used to infer gene regulatory network activities of individual NETS subtypes (Fig. 2E). A transcription factor motif analysis of these gene regulatory networks was executed to identify potential cell type–specific transcription factors (*34*). In keeping with their proposed differentiation from mesothelial cells, CapFb3 fibroblasts expressed gene regulatory networks controlled by the transcription factors *Hoxa5* and *Wt1*, which typically are active in mesothelial cells (Fig. 2E and fig. S4C) (*14*). CapFb4 was highly enriched for a *Creb5*-controlled gene regulatory network that has previously been identified to modulate the differentiation of fibroblasts to myofibroblasts (*35*) and to control age-related thymic fibrosis (fig. S4D) (*36*). MedFb2b expressed *Irf7* encoding the interferon regulatory factor 7 (IRF7), a master regulator of type I IFN secretion that interacts with Smad3 to regulate TGF-β signaling for collagen production (Fig. 2E and fig. S4E) (*37*).

We assessed the expression of canonical Wnt signaling transcripts and growth factors known to be important in thymic stromal interactions with thymocytes (fig. S6) (*38*). Several Wnt ligands displayed distinct expression patterns among cells of the NETS. For example, *Wnt4* transcripts were detected in mesothelium; *Wnt5a* in CapFb2b, CapFb3, and CapFb4; *Wnt6* in NCC; and *Wnt10b* in CapFb4. Wnt modulators were also highly expressed in particular nonepithelial stroma cells, including *Rspo1* in mesothelium and *Sfrp5* in NCC. Several cell subtypes of the NETS acted as prominent sources of key growth factors, including *Bmp4* transcribed in CapFb1c; *Bmp7* in CapFb4 and mesothelium; *Fgf10* in CapFb1a, CapFb1b, and CapFb1c; and *Tgfb1* in endothelial cells. In keeping with their role in regulating the extracellular matrix, fibroblast subtypes showed high expression of key extracellular matrix transcripts, with collagens (e.g., *Col1a2*, *Col3a1*, and *Col14a1*) primarily expressed in capsular fibroblasts, and laminins (*Lama2* and *Lama4*) in a mixture of capsular (CapFb1a, CapFb1b, and CapFb1c) and medullary fibroblasts (MedFb1b and MedFb2b). This distinctive expression of growth and differentiation factors, and components of the extracellular matrix, demonstrated that heterogeneity within the NETS compartment determined modularity in the expression of key molecules, thus implicating different developmental and functional niches.

### Ligand-receptor pairing analysis identifies interactions between neural crest–derived mesenchyme and endothelial cells

**Fig. 3.**
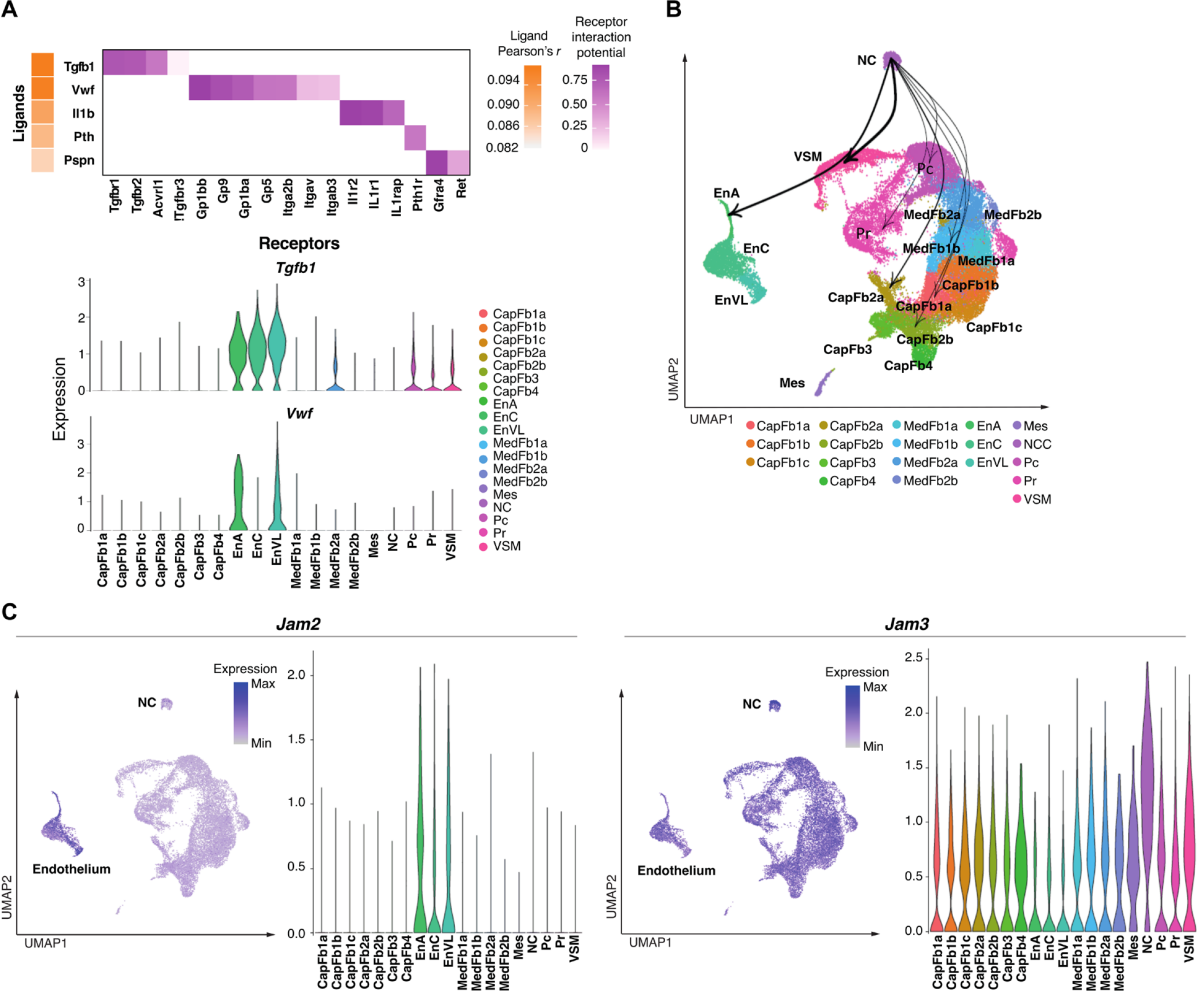
Ligand-receptor interaction of neural crest–derived cells and other thymic cell types. (**A**) Heatmap (top) of top ligand-receptor interactions of cell type signaling to NCC showing the Pearson’s *r* for ligand activity in promoting aging transition between E12.5/13.5 and E16.5. Violin plots (bottom) of the cell type–specific expression of the top two ligands, Tgfb1 and Vwf. (**B**) UMAP plot showing weighted connections for the top 25% ligand-receptor target networks for connections from neural crest cells. The widths of the lines are proportional to the strength of ligand-receptor interactions. (**C**) UMAP and violin plots of *Jam2* and *Jam3* expression.

Given that NCCs are known to differentiate into perivascular cell types, we aimed to uncover the ligand-receptor signaling and the subsequent transcriptomic networks that control the differentiation of NCCs into Pc and VSM (*7*, *31*). To this end, NicheNet identified intercellular ligand-receptor interactions associated with cell type–specific transitions across early (E12.5 and E13.5) to later stages (E16.5) in embryonic thymus formation (*39*). This analysis demonstrated that ligands expressed by endothelial cells, including the adhesive and multimeric glycoprotein von Willebrand factor (vWF) and transforming growth factor beta 1 (TGFB1), influenced gene expression in thymic NCC (Fig. 3, A and B). Conversely, heterotypic interactions between junctional adhesion molecule 3 (*Jam3)* produced by NCC and its receptor *Jam2* on endothelial cells identified a candidate ligand-receptor pair that orchestrated the changes in the gene expression profile of embryonic endothelial cells (Fig. 3C). Together, these inferred ligand-receptor interactions suggested that reciprocal cellular relationships between vascular structures and NCC shape the perivascular thymic stroma during embryogenesis.

### Reduced cellularity and complexity of mesenchymal stroma are features of an experimental 22q.11.2 deletion syndrome model

Heterozygous deletions of 1.5 to 3 Mb occur spontaneously within 22q11.2 due to recombination between four blocks of low copy repeats resulting in the loss of up to 106 genes (*40*). The 22q11.2 deletion syndrome (22q11.2DS) constitutes the most common molecular etiology of DiGeorge syndrome, which manifests clinically with a range of features that include either athymia resulting in T cell deficiency or thymus hypoplasia compromising immunological fitness (*40*). Regions of mouse chromosome 16 are syntenic to the human 22q11.2 (*41*) and include *Tbx1*, encoding a T-box transcription factor, and *Crkl*, encoding an adapter protein implicated in fibroblast growth factor and focal cell adhesion signaling (*40*). Compound haploinsufficiency of *Tbx1* and *Crkl* in mice (designated *Tbx1*^+/−^*Crkl*^+/−^) results in typical hallmarks of 22q11.2DS, including thymic hypoplasia (*42*).

The abnormal migration of cephalic NCC has been identified as a possible cause for the pharyngeal patterning defects observed in 22q11.2DS, which is recapitulated in *Tbx1*^+/−^*Crkl*^+/−^ mice. Gene products of these two loci have been alleged to interact in a dosage-sensitive fashion (*42*). *Tbx1* expression in NETS was exclusively confined to E12.5 and detected in a subset of cortical fibroblast subtypes, especially CapFb4, and proliferating cells. Yet, *Crkl* transcripts were mainly detected in CapFb1 and CapFb4 subtypes early during thymus development but could also be identified in a small fraction of these and other NETS later in development (fig. S7, A to C).

**Fig. 4.**
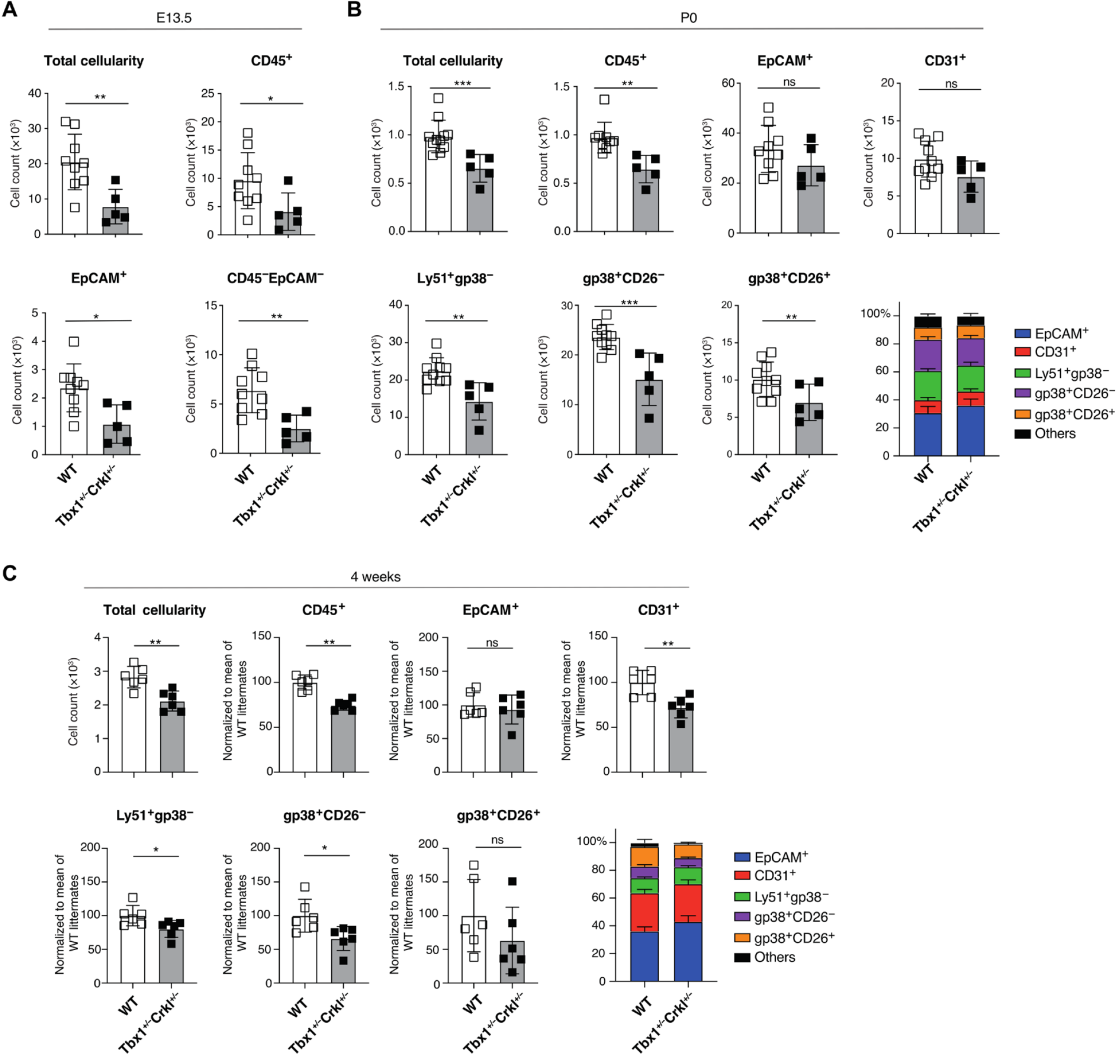
Reduction of nonepithelial thymic stroma in *Tbx1*^+/−^*Crkl*^+/−^ thymi. (**A**) Total cellularity, number of CD45^+^, EpCAM^+^, and total mesenchymal cells (CD45^−^EpCAM^−^) at E13.5 WT (white bars, *n* = 9) and *Tbx1*^+/−^*Crkl*^+/−^ (gray bars, *n* = 5) thymi. (**B**) Total thymic cellularity; absolute number of CD45^+^, EpCAM^+^, CD31^+^, Ly51^+^gp38^−^, gp38^+^CD26^−^, and gp38^+^CD26^+^ cells; and the frequency of stromal subpopulations in (B) neonatal (P0) and (**C**) 4-week-old WT (P0: *n* = 9, 4 weeks: *n* = 6) and *Tbx1*^+/−^*Crkl*^+/−^ (P0: *n* = 5, 4 weeks: *n* = 6) mice. Data were normalized to the mean of WT littermates from two independent experiments (*n* = 3 for each experiment). Mean values are shown in the bar charts. Unpaired *t* test, *P < 0.05, **P < 0.01, and ***P < 0.001. ns, not significant.

The thymi of mice compound heterozygous for a loss of *Tbx1* and *Crkl* were hypoplastic and revealed already at E13.5 significantly fewer hematopoietic, epithelial, and mesenchymal cells than their wild-type (WT) controls, which was not the case for either single *Tbx1* or *Crkl* mutants (Fig. 4, A to C, and fig. S7D). At birth, hematopoietic cells and all phenotypically identified major NETS subpopulations were reduced in mutant mice and remained diminished in 4-week-old mice, with the notable exception of gp38^+^CD26^+^ capsular fibroblasts. In contrast, the cellularity of TEC and endothelial cells varied over time and were not uniformly reduced in mutant mice at these times (Fig. 4, B and C, and fig. S7E). Thus, several NETS subpopulations were consistently reduced in *Tbx1*^+/−^*Crkl*^+/−^ mice.

**Fig. 5.**
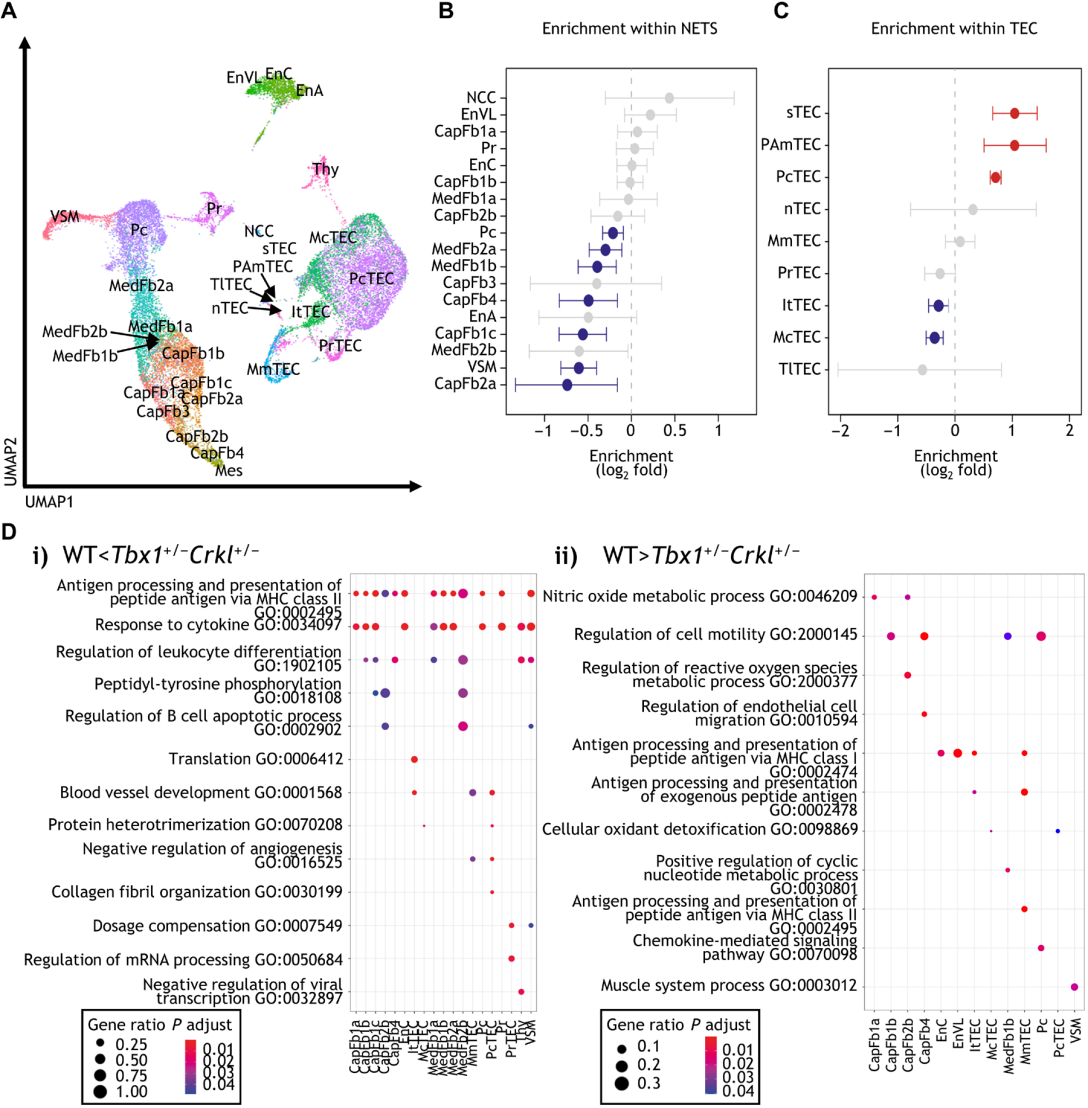
Thymic stromal cells from *Tbx1*^+/−^*Crkl*^+/−^ and wild-type mice differ transcriptomically within cellular populations. A total of 10,300 cells from *Tbx1^+/−^Crkl^+/−^* and 11,151 cells from their WT littermates were analyzed. (**A**) UMAP plot showing thymic stromal cell types. Scatter plot of genotype-specific enrichment of cell type frequency in *Tbx1*^+/−^*Crkl*^+/−^ and WT within NETS (**B**) and TEC (**C**). Error bars show 95% confidence intervals. (**D**) Gene ontology (GO) analysis of genes more highly expressed within each cell type in (i) *Tbx1*^+/−^*Crkl*^+/−^ and (ii) WT thymi. ItTEC, intertypical TEC; McTEC, mature cortical TEC; MmTEC, mature medullary TEC; nTEC, neural TEC; PAmTEC, post-AIRE medullary TEC; PcTEC, perinatal cortical TEC; PrTEC, proliferating TEC; sTEC, structural TEC; Thy, thymocyte; TlTEC, tuft-like TEC. Red symbols show cell subtypes significantly enriched in *Tbx1*^+/−^*Crkl*^+/−^, and blue symbols show subtypes enriched in WT (B and C). Enrichments were calculated using Fisher’s exact test with 95% confidence intervals, and significance was adjusted for multiple hypothesis testing using Benjamini-Hochberg correction (B and C).

We next compared the transcriptome of individual epithelial and NETS cells isolated from newborn *Tbx1^+/−^Crkl^+/−^* mice [Fig. 5A and fig. S8A; epithelial and nonepithelial stromal cells were annotated as previously published (*2*) and shown in Fig. 2, respectively]. *Tbx1* and *Crkl* compound heterozygosity substantially changed the composition of the thymus stroma resulting in a reduction of 8 of the 18 individual subtypes in *Tbx1^+/−^Crkl^+/−^* mice. Specifically and in contrast to the results obtained by flow cytometry, the relative cellularity of several capsular and medullary fibroblast subtypes together with that of Pc and VSM was lessened (Fig. 5B). In addition, the frequencies of mature cortical (mcTEC) and intertypical TEC (itTEC) were reduced in mutant mice, whereas those of perinatal cortical TEC (pcTEC), post-Aire mTEC (pamTEC), and structural TEC (sTEC) were enriched (Fig. 5C).

To assess the functional consequences of a compound heterozygous loss of *Tbx1* and *Crkl*, we performed an enrichment analysis for differentially expressed gene sets (Fig. 5D and fig. S8B). This analysis revealed that transcripts for gene products relevant for cell migration were reduced in several capsular and medullary fibroblast as well as in Pc. In VSM, fewer transcripts for contractile elements (e.g., *Myl6* and *Tpm2*; Fig. 5D) were observed.

### Compound *Tbx1* and *Crkl* heterozygosity is associated with accelerated aging of thymic mesenchyme

**Fig. 6.**
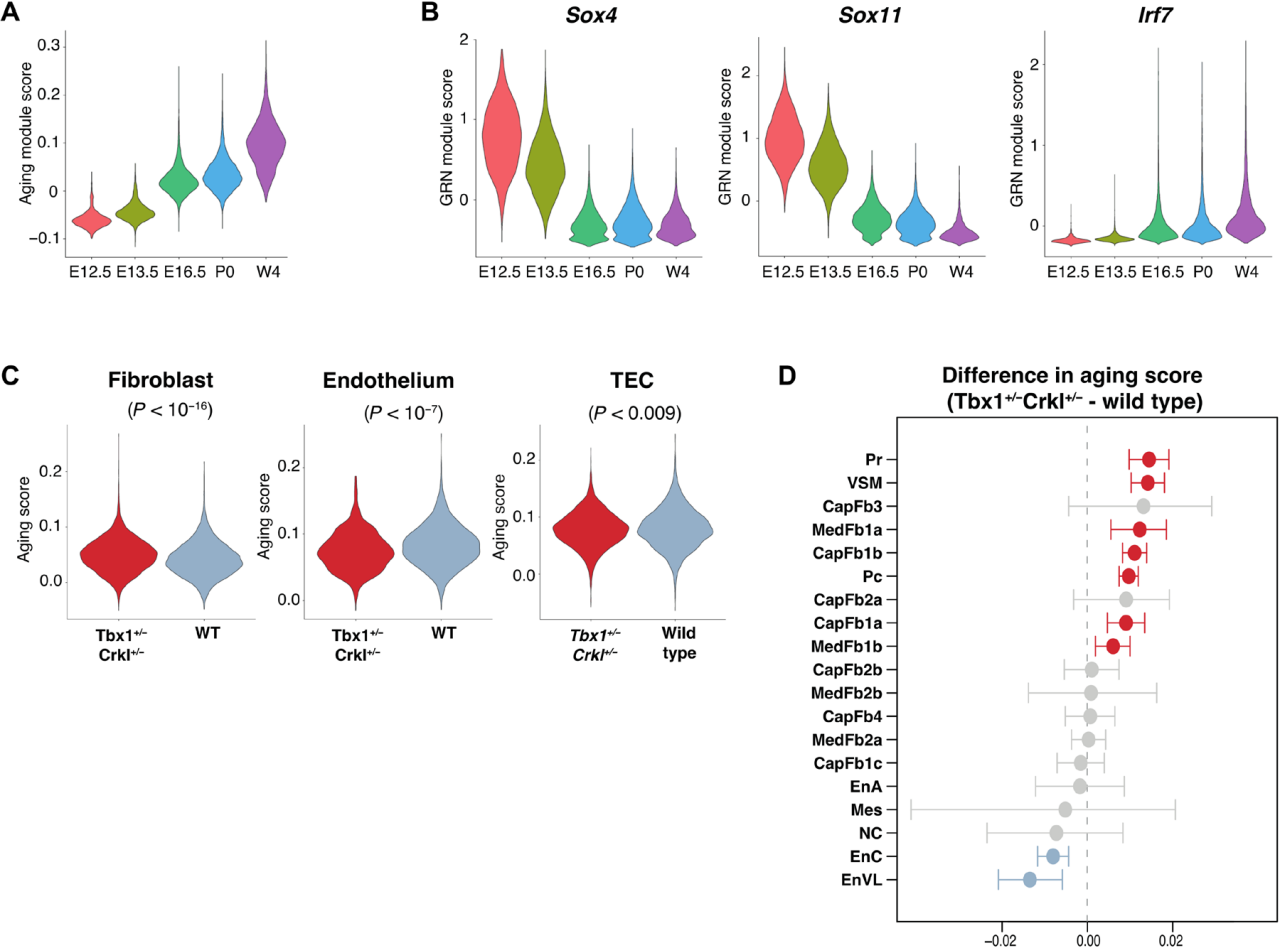
Age-specific transcriptomic programs differ over development in thymic stroma. (**A**) Violin plot showing the overall expression of genes associated with aging in a tissue-independent manner within the NETS across different ages (*45*). (**B**) Violin plots showing the expression of Sox4, Sox11, and Irf7 gene regulatory networks. (**C**) Violin plot showing the aging score of fibroblasts, endothelial cells, and TECs from *Tbx1*^+/−^*Crkl*^+/−^ and WT thymi at P0. (**D**) Enrichment plot of different NETS subtypes. Red symbols show subtypes with significantly increased aging scores in *Tbx1*^+/−^*Crkl*^+/−^ as compared to WT, and blue symbols show subtypes with significantly reduced aging scores in *Tbx1*^+/−^*Crkl*^+/−^. Differences in gene module scores were estimated using Wilcoxon tests and adjusted for multiple hypothesis testing using Benjamini-Hochberg correction (C and D).

Patients with 22q11.2DS display accelerated thymic senescence (*43*), a process thought to be caused by age-dependent chronic systemic inflammation (*44*). To appraise the effects of thymic senescence on NETS, we applied to our data an aging score computed from age-driven transcriptomic changes common across many tissues (*45*). In contrast to the heterogeneous effect of aging on TEC subsets (*2*), the aging module score of non-TEC stroma progressively increased from early embryonic stages to young adulthood (Fig. 6A). These changes demonstrated a switch from an abundant expression of transcripts belonging to biosynthetic pathways to gene products associated with angiogenesis and immunological cross-talk (fig. S9A).

Altered transcription factor network activity has been linked to the process of senescence (*45*). Using the transcriptomes from NETS isolated from mice at different ages, a decrease in transcripts related to gene networks controlled by SOX4 and its close relative SOX11 was observed. In parallel, gene networks controlled by IRF7 were gradually activated over time and beyond what would be expected from their enrichment in MedFb2b subsets (Fig. 6B and fig. S4E). In contrast, FOS and FOSB controlled gene networks peaked at birth but were subsequently weakened (fig. S9B), a pattern previously noted in other tissues (*46*).

The aging module score analysis was extended to include thymic stromal cells isolated from *Tbx1^+/−^Crkl^+/−^* mice at postnatal day 0 (P0). Accelerated aging (as discernible by an increased score) was noted in mutant mice for the population of mesenchymal but not endothelial and epithelial cells (Fig. 6C). Within the mesenchymal compartment, accelerated aging was not uniform, as an increased score was observed in only 7 of the 19 distinct stromal subtypes including Pc, VSM, and two cortical and medullary fibroblast subtypes (Fig. 6D). Hence, these studies showed age-related transcriptomic changes across distinct NETS subtypes of *Tbx1^+/−^ Crkl^+/−^* mice. Although there were only a very small number of NCCs present at P0 in either WT or *Tbx1^+/−^Crkl^+/−^* mice, NCC migration and differentiation are known to be impaired in 22q.11.2DS (*47*). It is therefore possible that the alterations within the NETS compartment observed in *Tbx1^+/−^Crkl^+/−^* mice may be driven by aberrant differentiation of NCCs into mesenchymal cells, particularly perivascular cell types (*31*).

### Neural crest cells differentiate into perivascular cells in the human prenatal thymus

**Fig. 7.**
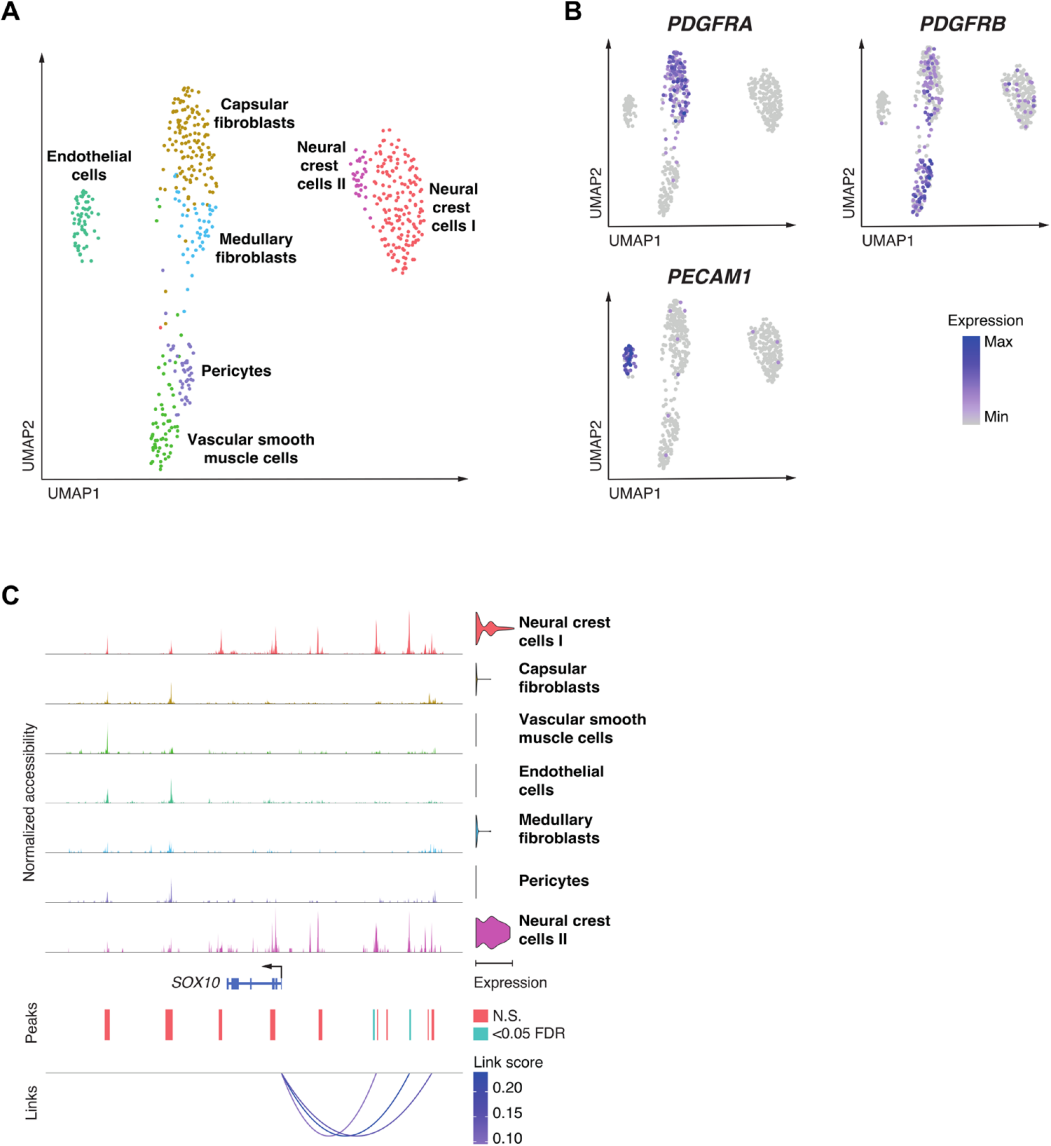
Single nuclei multiomics analysis of human thymic NETS demonstrates diverse cell populations. (**A**) UMAP plot of 528 nuclei showing joint projection of transcriptomic and chromatin accessibility data. (**B**) UMAP plot showing marker gene expression for *PDGFRA*, *PDGFRB*, and *PECAM1*. (**C**) Links plot of the *SOX10* locus showing chromatin accessibility data, *SOX10* gene expression (violin plot on right), accessible chromatin peaks [blue peaks were significant for at least one cell type at false discovery rate (FDR) < 0.05], and the correlations between chromatin accessibility and *SOX10* gene expression (shaded by the strength of each link). Significance of motif activity and chromatin accessibility was calculated using likelihood ratios, correcting for the size of chromatin accessibility libraries. *P* values were adjusted for multiple hypothesis testing using Benjamini-Hochberg correction.

To extend the analysis of the thymus stroma to human tissue, we used single NETS nuclei to investigate their gene expression profiles and correlated these to chromatin accessibility. For this purpose, we used a multiomics analysis that investigated 528 individual non-TEC thymic stroma nuclei isolated from two donors at 14 and 17 weeks after conception (figs. S10 and S11). This analysis identified seven distinct cell clusters, corresponding to NCCs (NCC-I and NCC-II), capsular fibroblasts, VSM, endothelial cells, medullary fibroblasts, and Pc. The frequency of cells in NCC-I increased from 14 to 17 weeks after conception, whereas the frequency of those in endothelium and Pc decreased within that time span (fig. S12). We found the expected gradients in *PDGFRA* and *PDGFRB* expression across clusters composed of fibroblasts, Pc, and VSM (Fig. 7A) and *PECAM1* expression in endothelial cells (Fig. 7B). As in the mouse, the NCCs showed highly specific expression of *SOX10*, whereas *SOX6* was expressed in both NCCs and VSM, and *SOX18* in endothelia (fig. S2).

Given the dynamic changes in NCC frequency in the thymus over development and the changes observed in NCC-derived perivascular structures in *Tbx1^+/−^Crkl^+/−^* thymi, we focused further on the chromatin and transcriptomic landscape of the NCC subclusters. NCC development is understood as a stepwise series of bifurcating cell fate decisions that lead to multiple cell identities and traits (*48*). Once specified in their fate, NCCs undergo an EMT and migrate throughout the embryo. NCC-II showed genome-wide high accessibility for sequences with SOX10 transcription factor binding motifs, whereas NCC-I displayed only an intermediate degree of accessibility for this motif (fig. S13).

To further investigate the dynamics of SOX10 activity, we integrated gene expression profiles with chromatin accessibility in single cells to identify key enhancer—transcriptional start site interactions driving *SOX10* expression in human NCCs (Fig. 7C). We identified two chromatin accessibility peaks, which were significantly correlated with *SOX10* expression. These peaks overlap two upstream orthologous enhancer elements (called U2 and U3) previously implicated in the control of *SOX10* expression in mouse NCCs and their progeny (*49*). Having identified that *SOX10* regulation was shared throughout the two NCC subclusters, we further examined heterogeneity among these NCCs to establish whether these could represent NCCs in different states of differentiation.

The comparative analysis of the gene expression profiles among the two NCC subclusters revealed an enrichment of gene pathways associated with cellular motility and vascular development for NCC-I (e.g., *BCL2*, *FGF13*, and *RHOJ*; fig. S14A). In contrast, NCC-II was enriched for gene pathways related to neuronal development and thus marked cells with gene expression profiles characteristic of bona fide NCC (e.g., *NES*, *NCAM2*, and *GRID2*; fig. S14A). This difference in gene expression profiles suggested that NCC-I may constitute a population of NCCs differentiating into mesenchymal and perivascular cell types. In support of this notion, NCC-II showed in comparison to NCC-I a significantly higher expression of *TFAP2A*, a key transcription factor in early NCC development, whereas *NR2F2*, a transcription factor involved in NCC migration, was most highly expressed in NCC-I (fig. S14B) (*50*). Hence, the multiomics analysis of NETS cells in human thymi captured the process of NCC differentiation into other cell types, a finding that could not be as clearly observed in mouse thymic stromal samples analyzed in this study despite similar levels of *SOX10* expression within the NCC compartment.

## DISCUSSION

Stromal cells with separate functions emerge from all germ layers during development to populate organs, where they instruct the tissue’s essential activities, for example, via the differential production of extracellular matrix components, the release of growth and differentiation factors, and the creation of signaling niches that provide critical molecular cues (*51*). In addition to cross-organ communalities, stromal cells with seemingly identical phenotypes also display a heterogeneity both within and across tissues as revealed by dissimilarities in transcripts encoding pathway elements, transporters, and cell surface markers (*52*). Previous studies of the thymic stroma in both mice and humans could identify only a limited number of phenotypically distinct thymic mesenchyme subtypes (*13*, *38*, *53*) despite the cells’ acknowledged roles as critical components in maintaining tissue structure and TEC function (*8*).

Using gene expression profiles at single-cell resolution, we now show an unprecedented heterogeneity among thymic mesenchymal cells and identify dynamic changes in the frequency of these cells across a large range of developmental stages. Notably, the observed diversity is not replicated using flow cytometry since transcriptionally defined stromal subtypes display identical phenotypic features due to a limited number of suitable cell surface markers. This limitation has hindered a comprehensive understanding of how nonepithelial thymic stromal cells contribute to local tissue microenvironments, which control discrete stages of intrathymic T cell differentiation.

Early in development, the thymus stroma is mostly composed of cells belonging to the NETS and, with the notable exception of NCC, continues to structure the scaffold also in adult mice where they contribute together with TEC to the nonhematopoietic stroma. We identify within the NETS separate cell types as decoded by unique transcriptional fingerprints, including endothelial cells, vascular mural cells, NCCs, mesothelial cells, and fibroblasts. Among the fibroblast population, at least 11 distinct capsular and medullary subtypes are recognized, thus largely extending the previously identified heterogeneity defined mostly by phenotypic markers and bulk RNA-seq (*13*, *38*, *53*). These subtypes display dynamic changes in their relative representation over time and demonstrate RNA splicing patterns that identify CapFb1a, CapFb2b, and MedFb1a as fibroblast subtypes with precursor potential and CapFb3 to originate from mesothelial cells, as this fibroblast subtype continues to expresses several mesothelium-specific biomarkers, including *Wt1*, *Cxcl13*, and *Rspo1* [fig. S15; (*14*)].

Cells with gene expression profiles typical of arterial, capillary, and venous vasculature are detected already at E12.5 when the colonization of the thymus by hematopoietic precursor cells has been initiated independent of an established vasculature (*54*). After E15.5, the frequency of Pc and VSM cells increases, which coincides with the histological evidence of vessel formation. The gatekeeper molecules P-selectin, ICAM-1, VCAM-1, and CCL25 enable the entry of T cell precursors into the thymic microenvironment, and we find these molecules expressed by all thymic endothelia in the postnatal thymus (*55*). The expression level of the adhesion molecules increases parallel to the age of the mouse but differs between distinct anatomical sites along the vasculature (fig. S16). This expression pattern specifies that T cell precursors enter the thymic microenvironment via postcapillary venules in a gated and temporally controlled way (*56*) and that the efficiency of this process may differ between developmental stages. The further development of these hematopoietic cells is regulated by membrane-bound Kit ligand, which we find expressed by all endothelia and thus also at those anatomical locations where hematopoietic precursors enter the thymus microenvironment (fig. S16).

Endothelial cells also regulate, in a nonredundant fashion, the egress of mature thymocytes via the expression of sphingosine-1-phosphate (S1P) lyase (encoded by *Sgpl1*), lipid phosphate phosphatase 3 (*Pllp3*), and spinster homolog 2 (*Spns2*) that modify S1P availability and enable the molecular transport, respectively (*57*–*59*). Transcripts for *Ppl3* and *Spns1* are detected in all endothelial cell types even paradoxically at a time of development when T cell export has not yet commenced (fig. S16). This expression pattern designates the anatomical site from where thymocytes can exit and endorses the molecular mechanism by which this process is controlled (*60*).

The NETS collectively promotes the proliferation and differentiation of TEC either indirectly via ligands that engage, for example, the platelet-derived growth factor receptor α (PDGFRα) (*61*) or directly via different signaling ligands, including Wnts, BMP4, Fgf7, and Fgf10 (*4*, *62*, *63*). For example, BMP is expressed by CapFb1c together with Fgf10 and up-regulates FOXN1, a transcription factor indispensable for TEC differentiation and function (*62*–*64*). Wnt4, which also stimulates the up-regulation of FOXN1 in both an autocrine and paracrine way, is not expressed by any of the identified thymic fibroblast subtypes but detected in mesothelial cells, thymocytes, and epithelia within the thymus (*63*, *65*). Moreover, three of the four capsular fibroblast subtypes express a range of Wnt ligands, albeit none that had previously been implicated in stimulating FOXN1 expression. The transcriptome of individual thymic fibroblast subtypes also infers that Wnt-mediated signals are furthermore either positively modulated by R-spondin 1 secreted by mesothelia or negatively delimited by Kremen and Dickkopf-1, which are expressed by other cellular components within the stroma (*66*, *67*). Moreover, CapFb4 fibroblasts, which express endosialin (*CD248*), have previously been implicated in the maintenance and regeneration of TEC (*68*).

A major feature of fibroblasts is their capacity to express extracellular matrix components, which form scaffolds that differ regionally in their composition, shape, biophysical characteristics, and functions (*51*). The heterogeneity and distinct spatial distribution of individual stromal cells therefore account for the diverse properties of the extracellular matrix, with collagen expression restricted to capsular fibroblasts and transcripts for laminins detected more widely across capsular and medullary fibroblast subtypes (fig. S6). Patterning of the extracellular matrix is critical in supporting thymic organogenesis, suggesting that thymic mesenchymal diversity will likely have a broad impact on thymic *in vivo* function (*9*).

Single-cell sequencing of human thymus tissue identifies a heterogeneity among NETS that is similar to the variance observed in mice and thus constitutes a trans-species phenomenon (*38*, *53*). Unexpectedly, a relatively large population of NCCs is still detected at 14 and 17 weeks after conception, i.e., at a time when thymus morphogenesis has ended and full function has been attained (*69*). This finding thus contrasts the results observed in mouse tissue at a corresponding developmental stage since the thymus of mice largely lacks NCCs as early as E16.5. This incongruity suggests that, contrary to mice, human NCCs have a more enduring role in shaping the NETS compartment. The analysis of human thymus tissue at late fetal stages of thymus organogenesis reveals two distinct NCC subtypes each with a seemingly different developmental potential, bespoke chromatin accessibility, and proliferation dynamics. One of the two NCC subtypes, i.e., NCC-I, represents a subtype that is poised to adopt a mesenchymal and perivascular cell fate. Notably, the corresponding cell type is not identified in the mouse thymus, although there is evidence of transcriptomic heterogeneity, albeit less clearly, within NCCs in mice as in humans (*33*). This suggests that the transition between NCC and perivascular cells in mice either must be rapid and profound or, alternatively, occurs at an embryonic time point that is not captured in our dataset, as the lineage mapping of NCCs has previously identified their differentiation into VSM and Pc (*7*, *31*).

The murine model of 22q11.2DS reveals major quantitative and qualitative changes in the thymus stroma, which are highlighted by a decreased proportional representation of several TEC and mesenchymal subtypes, the latter including a selection of capsular and medullary fibroblasts and the NCC-derived Pc and VSM. However, *Tbx1* and *Crkl* transcripts are only solidly detected in a selection of mouse thymus fibroblast subtypes at the earliest stages of organogenesis and are notably absent in thymic NCCs. NCCs retain their relative frequency in the presence of compound *Crkl* and *Tbx1* haploinsufficiency. Hence, the observed modifications in vascular mural cells are not the result of a reduced thymic NCC frequency or changes in *Crkl*- and *Tbx*-controlled gene expression in these cells having migrated to the thymus. Rather, our data suggest that they are the consequence of altered TGF-β receptor–mediated signaling in Pc, VSM, and likely their immediate precursors resulting in altered CRKL phosphorylation, with *Tgfb1* expressed by endothelial cells and *Tgfb3* by capsular fibroblasts (fig. S17) (*70*). Yet, the absence of normal CRKL-dependent signaling in different haploinsufficient fibroblast subtypes may additionally and indirectly impair Pc and VSM development, thus arguing for a hitherto unexplored aspect of intrathymic cellular cross-talk.

It is thought that alterations in NETS cell type abundance have an impact on TEC function. *Tgfb1* is a key ligand predicted to drive the transcriptomic differences underlying *Tbx1* and *Crkl* haploinsufficiency in perinatal cTEC (fig. S17). These transcriptomic differences included significantly lower expression of FOXN1 target genes in *Tbx1^+/−^Crkl^+/−^* relative to WT perinatal cTEC (*P* < 0.0001, Wilcoxon rank sum test) (*64*).

Overall, our findings have identified previously underappreciated levels of cellular heterogeneity and developmental dynamics within the non-TEC thymic stromal compartment. Cellular diversity within this compartment is present in both murine and human thymic development but shows clear trans-species differences worthy of further investigation. Many of these cell populations are disrupted in 22q11.2DS, a syndrome known to cause defective thymic organogenesis and function. Further work should focus on identifying the precise function of each fibroblast cell subpopulation, along with their contribution to overall thymic development and function.

## MATERIALS AND METHODS

### Mice

All mice were maintained under specific pathogen–free conditions and according to United Kingdom Home Office regulations and federal regulations and permissions, depending on where the mice were housed. WT C57BL/6 mice originated were bred in-house. A mouse line carrying a germline *Crkl* null allele (Crkl^tm1d(EUCOMM)Hmgu^/ImoJ) was generated with Cre-mediated recombination in the epiblast by crossing the *Crkl*-flox mice (Crkl^tm1c(EUCOMM)Hmgu^/ImoJ) (*71*) with Meox2 Cre knock-in strain (*72*), followed by backcrosses with WT C57BL/6 mice to segregate out Meox2Cre. Tbx1^lacz/+^ (*73*) mice were obtained from A. Baldini via P. Scambler at University College London. *Tbx1* and *Crkl* compound heterozygous mice (*Tbx1*^+/−^*Crkl*^+/−^) were generated by crossing between *Tbx1*^+/−^ males with *Crkl*^+/−^ females. Embryos of specific embryonic ages were obtained through timed mating, where the presence of vaginal plug was defined as E0.5.

### Isolation of mouse thymic stromal cells and preparation for flow cytometry

Thymic cell suspensions were obtained via enzymatic digestion of thymic lobes using Liberase (Roche) and deoxyribonuclease (DNase) I (Roche). To enrich for nonhematopoietic stromal cells in thymic digests from adult mice, cell suspensions were counted and stained with anti-CD45 microbeads (Miltenyi Biotec) for 15 min on ice, before negative selection using the AutoMACS (Miltenyi Biotec) system. Enriched samples or nonenriched samples were then stained for cell surface markers for 30 min at 4°C. For intracellular staining, the Foxp3 Transcription Factor Staining Buffer Kit (eBioscience) was used according to the manufacturer’s instructions. Combinations of UEA-1 lectin (Vector Laboratories) labeled with BV605 and the following antibodies were used to stain the cells: TER-119::BV421 (BioLegend), CD45::AF700 (30-F11, BioLegend), EpCAM::PerCPCy5.5 (G8.8, BioLegend), Ly51::PE (6C3, BioLegend), CD80::PECy5 (16-10A1, BioLegend), CD26::PECy7 (H194-112, BioLegend), MHCII::APCCy7 (M5/114.15.2, BioLegend), MHCII::BV421 (M5/114.15.2, BioLegend), CD31::AF488 (MEC13.3, BioLegend), and podoplanin (gp38)::AF647 (PMab-1, BioLegend). 4′,6-Diamidino-2-phenylindole (DAPI) or the LIVE/DEAD Fixable Aqua Dead Cell Stain Kit was used (Thermo Fisher Scientific) for the assessment of cell viability. After staining, cells were acquired and sorted using FACSAria III (BD Biosciences) and analyzed using FlowJo v10 and GraphPad Prism 8. Statistical analyses were performed using *t* tests, with correction for multiple comparisons where appropriate. A *P* value or the adjusted *P* value of ≤0.05 was considered statistically significant.

### Immunofluorescent microscopy for extracellular matrix proteins

Thymus from a 5-week-old female WT C57BL/6J mouse was used. The standard procedure for immunofluorescence on tissue sections was described here (www.biorxiv.org/content/10.1101/2021.03.21.436320v1). Briefly, organs are collected in phosphate-buffered saline (PBS) and fixed in 4% paraformaldehyde overnight at 4°C on a rotating shaker. Organs were then washed in PBS, and lobes were separated for the next steps. Paraffin infiltration was done using a Tissue-Tek VIP 6 AI Vacuum Infiltration Processor (Sakura). Lobes were then embedded in paraffin, and 4-μm sections were cut with a Leica RM2265 microtome.

Before immunostaining, dewaxing and antigen retrieval in citrate buffer at pH 6.0 (using a heat-induced epitope retrieval PT module, Thermo Fisher Scientific) were performed. Sections were then blocked and permeabilized for 30 min in 1% bovine serum albumin (BSA) and 0.2% Triton X-100 in PBS and blocked for 30 min in 10% donkey serum (Gibco) in PBS at room temperature (RT). Sections were incubated with primary antibodies overnight at 4°C in 1.5% donkey serum in PBS. Sections were washed twice in 1% BSA and 0.2% Triton X-100 in PBS and incubated with secondary antibodies at RT for 45 min. Last, sections were washed twice in 0.2% Triton X-100 in PBS and mounted with Fluoromount-G (SouthernBiotech). Pictures were acquired with a charge-coupled device DFC 3000 black and white camera on an upright Leica DM5500 scanning microscope. Image processing only included brightness and contrast adjustments in Fiji/ImageJ.

The following antibodies were used: goat anti-fibronectin (Santa Cruz Biotechnology, sc-6953, 1:250), rabbit anti–collagen 1 (Abcam, ab21286, 1:250), donkey anti-goat Alexa Fluor 488 (Thermo Fisher Scientific, A-11055, 1:500), and donkey anti-rabbit Alexa Flour 647 (Thermo Fisher Scientific, A-31573, 1:500). For nuclei staining, DAPI (Sigma-Aldrich, 1 μg per ml) was used.

### Immunofluorescent microscopy for CD26 and podoplanin

Freshly isolated thymic lobes were frozen in optimal cutting temperature (OCT) compound (Tissue-Tek) and cryosectioned at a thickness of 10 μm. Tissue sections were fixed with ice-cold acetone for 5 min and blocked with the Avidin/Biotin Blocking Kit (Vector Laboratories) and Protein Block (Dako) according to the manufacturer’s protocol. Tissue sections were then incubated with primary antibodies at 4°C overnight: rabbit anti-mouse CD26 (DPP4) [EPR5883(2), Abcam] and biotin anti-mouse podoplanin (8.1.1, BioLegend). Secondary antibody staining was performed at RT for 30 min with anti-rabbit:AF488 (Invitrogen) and streptavidin-AF555 (Invitrogen). Nuclei were stained with Hoechst 34580 in PBS (according to the manufacturer’s protocol). Sections were mounted with ProLong Gold Antifade Mountant (Thermo Fisher Scientific) and acquired using an LSM700 confocal microscope (Carl Zeiss AG). Image analysis was performed with ImageJ software (W. S. Rasband, ImageJ, U.S. National Institutes of Health, Bethesda, MD).

### RNA in situ hybridization and imaging

The RNAscope Multiplex Fluorescent V2 assay (Bio-Techne, catalog no. 323110) was performed according to the manufacturer’s protocol on 4-week-old female WT C57BL/6J thymus paraffin sections (see the “Immunofluorescent microscopy for extracellular matrix proteins” section for organ preparation before sectioning). Sections were cut at 3 μm with a Leica RM2265 microtome and hybridized with the probes Mm-Csmd1 (Bio-Techne, catalog no. 444791) or Mm-Pi16-C3 (Bio-Techne, catalog no. 451311-C3). Mm-3Plex probes (Bio-Techne, catalog no. 320881) and 3Plex Dapb probes (Bio-Techne, catalog no. 320871) were used as positive and negative controls, respectively. Probes were incubated at 40°C for 2 hours, and the different channels were revealed with TSA Opal570 (Akoya Biosciences, catalog no. FP1488001KT) and TSA Opal650 (Akoya Biosciences, catalog no. FP1488001KT). Tissues were counterstained with DAPI and mounted with ProLong Gold Antifade Mountant (Thermo Fisher Scientific, P36930). Images were acquired on a Leica SP8 STED 3X microscope with the following sequences: sequence 1: laser, 550 nm; filter, 560 to 610 nm; sequence 2: lasers, 405 and 627 nm; filters, 415 to 475 nm and 637 to 687 nm. Fluorescent signal was time-gated from 0.5 to 4 ns and acquired with hybrid detectors. Image processing only included brightness and contrast adjustments in Fiji/ImageJ for clearer visualization of the labeled cells.

### Single-cell RNA sequencing

Total thymic nonepithelial stromal cells (live Ter119^−^CD45^−^EpCAM^−^) from E12.5, E13.5, E16.5, P0, and 4-week-old WT mice were sorted and kept on ice before they were counted. A total of 18,000 cells per sample were loaded onto Chromium Single Cell B Chip (10x Genomics) followed by library preparation using Chromium Single Cell 3′ solution (10x Genomics) and sequencing by NovaSeq 6000 (28+98) (Illumina). For the Tbx1^LacZ/+^Crkl^+/−^ dataset, total non-hematopoietic stromal cells (live Ter119^−^CD45^−^) from P0 Tbx1^LacZ/+^Crkl^+/−^ (*n* = 3) and their WT littermates (*n* = 3) were sorted and fixed using RNAprotect Cell Reagent (Qiagen) for storage before sample submission to the Oxford Genomics Centre, where all downstream steps were performed including 10x Genomics Chip loading, library preparation, and sequencing.

### Single-cell RNA sequencing analysis

Sequencing reads were processed using Cell Ranger (version 3.1.0). Cells were retained for downstream analysis if there was expression of >1000 genes, <5% of unique molecular identifiers mapped to mitochondrial genes, cells were called as singlets by DoubletFinder, and cells did not cluster into *Ptprc* (CD45)–expressing clusters or other contaminant clusters (such as TECs or clusters present only in one replicate) (*74*). Seurat was used to remove batch effect between samples using canonical correlation analysis–based integration (*75*). Cells were projected into two-dimensional space using UMAP. Clusters were called using a resolution of 0.8, and cell label transfer between datasets was undertaken using Seurat. Differential analysis between clusters used Wilcoxon rank sum testing and over different ages used the Kruskal-Wallis analysis of variance. *P* values were corrected for multiple hypothesis testing using the Benjamini-Hochberg method. GENIE3 and RcisTarget were used to identify gene regulatory networks on highly variable genes expressed in at least 5% of cells, with subsequent module expression calculated using Seurat (*34*). RNA velocity analysis was undertaken using Velocyto and scVelo (*76*, *77*). Ligand-receptor target networks were inferred using NicheNet, with differential expression assessed between early (E12.5/13.5) and late (E16.5) embryogenesis (*39*). Gene ontology analysis was undertaken using clusterProfiler (version 4.0.0) (*78*).

### Single nuclei multiomics of human fetal thymic stroma

Human fetal thymi, obtained from terminations of pregnancy at 14 and 17 post-conception weeks, were enzymatically dissociated using Liberase (Roche) and DNaseI (Roche). The resultant cell suspension was stained with the following antibodies directed against cell surface antigens for 30 min at 4°C: CD45::BV421 (BioLegend, H130) and HLA-DR::PE-Cy7 (BioLegend, L243); 7-aminoactinomycin D (BioLegend) was used as a viability marker. Live CD45^−^ MHCII (major histocompatibility complex class II) intermediate-high cells were sorted in 250,000 cell aliquots using FACSAria III (BD Biosciences). Samples were then processed using the 10x Genomics Multiomics ATAC (Assay for Transposase-Accessible Chromatin using sequencing) + Gene Expression Kit according to the manufacturer’s protocol with some adaptations. Specifically, nuclei were isolated using a 0.1× diluted nuclei extraction buffer for 6 min before being captured into droplets on the 10x Genomics Chromium platform and sequenced on an Illumina NovaSeq machine. This study of human thymic tissue has been granted ethical approval and is publicly listed (IRAS ID 156910, CPMS ID 19587).

### Multiomics analysis

Sequencing data were processed using Cell Ranger ARC (version 1.0.1). Counts and ATAC data were analyzed using Seurat (version 4.0.3) and Signac (version 1.2.1) (*75*, *79*). Barcodes were filtered to high-quality cells (ATAC library size, 1000 to 100,000; RNA library size, 1000 to 31,622; ATAC peaks 1000 to 31,622; RNA features, 1000 to 10,000; and proportion of mitochondrial RNA reads, ≤0.15). ATAC peaks were recalled across each sample for all cells. Clusters were called on integrated RNA data using a clustering threshold of 0.8 and projected onto a joint UMAP plot of RNA and ATAC components generated using Seurat and Signac. Differential gene expression between clusters was estimated using the default method in Seurat. Differentially accessible peaks were identified using the likelihood ratio method with correction for ATAC library size. Motif activity was estimated using chromVAR with the JASPAR2020 motif dataset (*80*). RNA-ATAC links were analyzed using Signac and Seurat in 50-kb windows around genes of interest.

## Notes

View/request a protocol for this paper from *Bio-protocol*.
